# Microscopic Evidence of Malaria Infection in Visceral Tissue from Medici Family, Italy

**DOI:** 10.3201/eid2906.230134

**Published:** 2023-06

**Authors:** Frank Maixner, Dennis Drescher, Giulia Boccalini, Dario Piombino-Mascali, Marek Janko, Nicole Berens-Riha, Bum Jin Kim, Michelle Gamble, Jolanthe Schatterny, Rory E. Morty, Melanie Ludwig, Ben Krause-Kyora, Robert Stark, Hyun Joo An, Jens Neumann, Giovanna Cipollini, Rudolf Grimm, Nicole Kilian, Albert Zink

**Affiliations:** Eurac Research, Bolzano, Italy (F. Maixner, G. Boccalini, G. Cipollini, A. Zink);; University Hospital Heidelberg, Heidelberg, Germany (D. Drescher, J. Schatterny, R.E. Morty, M. Ludwig, N. Kilian);; Vilnius University, Vilnius, Lithuania (D. Piombino-Mascali);; Technical University of Darmstadt, Darmstadt, Germany (M. Janko, R. Stark);; Institute of Tropical Medicine, Antwerp, Belgium (N. Berens-Riha);; University of Munich, Munich, Germany (N. Berens-Riha);; Oregon State University, Corvallis, Oregon, USA (B.J. Kim);; Chungnam National University, Daejeon, Korea (B.J. Kim, H.J. An);; Heritage and Archaeological Research Practice, Edinburgh, Scotland, UK (M. Gamble);; Kiel University, Kiel, Germany (B. Krause-Kyora);; Ludwig Maximilian University, Munich (J. Neumann);; Agilent Technologies, Santa Clara, California, USA (R. Grimm)

**Keywords:** malaria, Medici, *Plasmodium falciparum*, immunohistochemistry, atomic force microscopy, parasites, vector-borne infections, zoonoses, Italy

## Abstract

Microscopy of mummified visceral tissue from a Medici family member in Italy identified a potential blood vessel containing erythrocytes. Giemsa staining, atomic force microscopy, and immunohistochemistry confirmed *Plasmodium falciparum* inside those erythrocytes. Our results indicate an ancient Mediterranean presence of *P. falciparum*, which remains responsible for most malaria deaths in Africa.

The Medici family was a powerful family from Florence, Italy, that gained prominence under Cosimo de’ Medici in the early 15th century (*1*). Dynastic power granted Medici family members a burial at the San Lorenzo Basilica in central Florence (Appendix Figure 1, panel A). Burial was preceded by an embalming procedure in which inner organs (viscera) were removed and placed in large terracotta jars (Appendix Figure 1, panel B).

In 2011, selected jars of organs from Medici family members were opened centuries after burial to examine their contents, revealing that multiple tissue pieces were still present (Appendix). The Institute for Mummy Studies at Eurac Research (Bolzano, Italy) received samples from the organs; we performed microscopic and molecular analysis (Appendix) of a 2.5 cm × 1.5 cm tissue piece (ID 1297) from 1 jar (Appendix Figure 1, panel C). Using microscopy, we identified a potential blood vessel containing erythrocytes (Figure, panel A). Diameters (7.24, SD ±0.14 µm; n = 37) and discocyte shapes of cells within the blood vessel were characteristic of erythrocytes (*2*). We conducted further microscopic evaluation of single cells and found the potential presence of a parasite that might have resided within the erythrocytes during the lifetime of the deceased family member. Giemsa staining of tissue sections confirmed our first impression (Figure, panel B) and suggested the parasite was *Plasmodium* spp.; members of this genus are the causative agent of different types of human malaria (*3*). We used atomic force microscopy to identify the ring stage, an immature developmental stage of *P. falciparum* that is dominant in peripheral blood of infected patients and a diagnostic hallmark (Figure, panel C). We verified the presence of *P. falciparum* by using immunohistochemistry with polyclonal mouse antiserum against *Plasmodium* spp.–specific aldolase (Appendix Figure 1, panels D, E) and a monoclonal antibody against *P. falciparum*–specific histidine-rich protein HRPII (Appendix Figure 1, panels F, G). We confirmed results by using immunofluorescence analysis with antibody against *P. falciparum* endoplasmic reticulum resident protein Pf39 (Appendix Figure 1, panels H, I). All isotype controls were negative (Appendix Figure 1, panels E, G, I). Not all observed parasitized erythrocytes were labeled by the antiserum, likely because of tissue degradation over the centuries. We verified a progressed state of biomolecule degradation by additional DNA-based analysis. 

**Figure:**
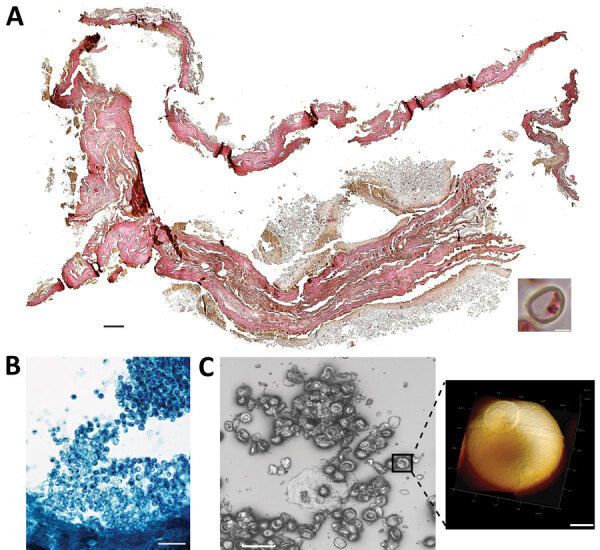
Microscopic analysis of malaria infection in visceral tissue from Medici family, Italy. We evaluated a 2.5 cm × 1.5 cm tissue piece (ID 1297) from 1 jar containing viscera of a Medici family member and identified a potential blood vessel containing erythrocytes. A) Histological cross section of the tissue stained with hematoxylin and eosin; scale bar indicates 200 µm. Inset shows a possible erythrocyte; scale bar indicates 3 µm. B) Giemsa staining of a paraffin section of viscera suggesting the presence of parasites within the erythrocytes. Scale bar indicates 50 µm. C) Atomic force microscopy (AFM) of the tissue section. An optical microscope was used to define appropriate sample areas for AFM imaging (left image); scale bar indicates 20 µm. Enlarged area at right shows a ring stage of *Plasmodium* *falciparum* in an erythrocyte; scale bar indicates 2 µm.

We determined that parasitemia was 38% in the Medici tissue, which appeared high (Appendix Figure 1, panel J). However, instead of peripheral blood, we investigated tissues that might have had higher than expected parasitemia from sequestration of erythrocytes parasitized by mature asexual developmental stages (trophozoite and schizonts) of *P*. *falciparum* (*4*). Erythrocytes were visible in the tissue and were not washed away after embedding, further suggesting the presence of malaria parasites because they can trigger blood coagulation that might have kept the cells in place (*5*). High parasitemia within tissues is likely dependent on *P. falciparum* developmental stages (*4*). Erythrocytes infected with juvenile ring stages can be found in the peripheral blood of patients, whereas mature developmental stages are absent (*6*). Erythrocytes that contain more mature developmental stages can adhere to endothelial cells that line blood vessels within inner organs (*6*).

The most striking parasite-derived erythrocyte modification is the establishment of secretory organelles, known as Maurer’s clefts, that reside within the cytoplasm of terminally differentiated host erythrocytes infected with *P. falciparum* (*7*). Similar organelles also exist in the cytoplasm of erythrocytes infected by other pathogenic *Plasmodium* spp. (*7*). During *P. falciparum* infections, Maurer’s clefts are crucial for initiating host-parasite interactions; they are responsible for severe disease and patient death by enabling protein trafficking that causes cytoadherence within organs (*4*). By using Giemsa staining, we observed delicate stipplings within the cytoplasm of infected erythrocytes in the Medici tissue, indicative of Maurer’s clefts (Appendix Figure 1, panel J). We quantified the stipplings; numbers were comparable to what can be observed within infected erythrocytes of malaria patients and in vitro–infected erythrocyte cultures.

We performed glycan analysis by using mass spectrometry and molecular analyses (Appendix). We identified a unique glycan found in erythrocyte B antigen (Appendix Figure 2, panels A–D), further indicating the presence of erythrocytes in the tissue. However, parasite DNA was undetectable by PCR. Metagenomic sequencing showed only 0.06% of all reads were host DNA; 2 reads could be unambiguously assigned to *P. falciparum* (Appendix Figure 2, panel E). 

Medici family members were known to hunt in marshlands around Florence and in Tuscany that served as breeding grounds for mosquito vectors capable of transmitting *Plasmodium* spp. parasites (*8*). In 2010, immunoassays were used to analyze bones of 4 Medici family members who might have died from malaria; *P. falciparum* was detected (*9*). Our observations agree with previous studies of ancient human remains, suggesting a Mediterranean presence of malaria from the era of ancient Egypt to modern times (*10*). Malaria remains a major health threat for persons in Africa, mostly affecting pregnant women and children. Malaria is a curable disease; however, persons in malaria-endemic areas still lack access to proper healthcare. Developing *Plasmodium* resistance to standard treatments further hampers positive therapeutic outcomes. 

Appendix Additional information for microscopic evidence of malaria infection in visceral tissue from Medici family, Italy.
