# Supplementary material for: Microscopic Evidence of Malaria Infection in Visceral Tissue from Medici Family, Italy
Source: Emerg Infect Dis. 2023 Jun;29(6):1280–3. doi: 10.3201/eid2906.230134 (PMC10202861; doi:10.3201/eid2906.230134)
Supplement: Appendix — Additional information for microscopic evidence of malaria infection in visceral tissue from Medici family, Italy. [file 23-0134-Techapp-s1.pdf]

Article DOI: https://doi.org/10.3201/eid2906.230134

EID cannot ensure accessibility for supplementary materials supplied by authors. Readers who have difficulty accessing supplementary content should contact the authors for assistance.

Microscopic Evidence of Malaria Infection in Visceral Tissue from Medici Family, Italy

Appendix

Additional Methods

Sampling Survey and Paleohistological Analysis

During a sampling survey in Florence, Italy, in 2011, soft tissue material was taken from Medici jars for further microscopic and molecular analysis. The samples were then transferred to the ancient DNA laboratory at Eurac Research, Institute for Mummy Studies, where the specimens were inventoried and subjected to further investigation. Initially, soft tissue samples collected from the jars were processed for histological analyses in the Eurac laboratory. Specifically, small soft tissue fragments (0.25 cm × 0.25 cm) underwent processing as previously described (*1*). After rehydration with Eurac solution (1:1 solution of glycerol and 4% formaldehyde) for 48 h, the samples were fixed for 24 h in 4% formaldehyde, dehydrated, and finally embedded in paraffin blocks. The embedded specimens were cut on a Leica RM2245 microtome (Leica Biosystems, https://www.leicabiosystems.com) into 4 µm-thick sections. Paraffin sections were stained with hematoxylin and eosin or Giemsa stains (*2*).

Staining of Paraffin Sections with Phosphate-Buffered Giemsa to Visualize Maurer's Clefts

The slides with paraffin-embedded tissues were positioned in a staining dish and immersed 2× in pure xylene for 5 min each. Then, they were placed consecutively in the following ethanol solutions: 100% (for 30 s), 96% (for 30 s), 96% (for 60 s), and 70% (for 60 s). Afterward, the slides were placed in double distilled (dd)H_2O for 2 min and subsequently incubated with Giemsa staining solution (10% Giemsa in phosphate buffer, pH 6.8) for 7 min at 60°C. The slides were placed again in ddH_2O for 1 min and subsequently dipped 1–4× in differentiating solution (50 mL ddH_2O and 5 drops of acetic acid). The slides were then

immersed in ddH_2O and successively dipped in solutions of 70%, 96%, 96%, and 100% ethanol (solutions obtained by diluting 100% ethanol with ddH_2O when necessary), followed by a brief dip into pure xylene. Finally, coverslips were placed onto the slides by using EUKITT mounting medium.

Atomic Force Microscopy

Tissue sections were examined by using a combined inverted Axiovert 135 optical microscope (Zeiss, https://www.zeiss.com) and NanoWizard-II atomic force microscope (AFM) (JPK Instruments/Bruker, https://www.bruker.com). The optical microscope was used to define appropriate sample areas for AFM imaging. AFM measurements were subsequently performed to obtain high-resolution images of the samples. AFM images were captured in the intermittent contact mode under ambient conditions. BS Tap300 silicon cantilevers (Budget Sensors, https://www.budgetsensors.com) with typical spring constants of 40 Newtons/m and nominal resonance frequencies of 300 kHz were used. The nominal tip radius was <10 nm. Images were analyzed by using SPIP 4.5.2 software (Image Metrology, https://www.imagemet.com).

Immunohistochemistry

All immunohistochemical staining was performed on 5 μm whole standard sections of formalin-fixed paraffin-embedded tissue samples. After deparaffinization, the slides were depigmented for 20 h in 1% HCl-alcohol at 37°C. Thereafter, the slides were washed in TRIS buffer 2× for 5 min each. The endogeneous peroxidase was blocked by placing the slides in 7.5% H_2O_2 for 19 min, followed by washing for 10 min under running tap water. Slides were rinsed consecutively 2× in TRIS buffer for 5 min each. For the detection of aldolase, an antibody against *Plasmodium* aldolase (Abcam, https://www.abcam.com) was used, or, for HRPII detection, *P. falciparum* HRPII monoclonal antibody (Thermo Fisher Scientific, https://www.thermofisher.com) was used as the primary antibody. The slides were subjected to heat-induced epitope retrieval by using Dako Target Retrieval Solution, Citrate pH6 (Agilent Technologies, https://www.agilent.com). Sections were incubated with primary antibodies (aldolase antibody dilution of 1:100; HRPII antibody dilution of 1:600) for 1 h at room temperature. The slides were then washed 2× in TRIS buffer for 5 min, and subsequently developed for 30 min by using ImmPRESS Reagent Kit Anti-MOUSE Ig (Vector Laboratories, https://www.vectorlabs.com). The slides were washed again 2× in TRIS buffer for 5 min each.

The Dako AEC+ system (Agilent Technologies) was used as chromogen. After washing for 10 min under running tap water, the slides were then counterstained with Gill´s formula hematoxylin (Vector Laboratories). To exclude non-specific staining, system controls were included.

Immunofluorescence Analysis

The immunofluorescence assay was conducted in accordance with the Beers Lab immunofluorescence protocol for paraffin sections from TLRC Heidelberg as follows. For deparaffinization, slides with formalin-fixed paraffin-embedded tissue sections were dipped 2× for 5 min each in xylene. Then, they were placed consecutively in 100% ethanol for 30 s and 96% ethanol for 30 s (solution obtained by diluting 100% ethanol with ddH_2O). Thereafter, the slides were placed successively in 96% ethanol for 60 s and 70% ethanol for 60 s (solutions obtained by diluting 100% ethanol with the corresponding amount of ddH_2O). The slides were finally placed in ddH_2O for 2 min. For antigen retrieval, a water bath containing sodium citrate buffer (10 mM sodium citrate, 0.05% Tween 20, pH to 6.0 with HCl; preparation: 1.47 mg Tris-sodium citrate dehydrate dissolved in 500 mL ddH_2O, and subsequent addition of 0.5 mL Tween 20) was heated to 95°C–100°C. Then, the slides with tissues were positioned in a staining dish, immersed in the hot sodium citrate buffer and incubated for 20 min. Afterward, the slides were removed from the buffer and cooled for 20 min at room temperature. For the final immunofluorescence staining, the slides were washed for 5 min in 0.05 mol/L phosphate-buffered saline (PBS;14.2 g Na_2HPO_4, and 16.0 g NaCl dissolved in 1800 mL ddH_2O, pH 7.2), and the tissue sections were subsequently flooded entirely for 3 min with $NaBH_4$ solution (10 mg/mL $NaBH_4$ in 0.05 mol/L PBS, prepared immediately before use). Then, the slides were washed in PBS containing 5% bovine serum albumin (BSA) for 5 min. The slides were rewashed in 0.05 mol/L PBS, flooded again with $NaBH_4$ solution, and washed with PBS/5% BSA. Afterward, the slides were covered entirely with $\geq$200 µL blocking buffer and incubated for 30 min. The blocking buffer was composed of 150 µL serum from the Vectastain Elite ABC Kit containing rabbit IgG or peroxidase rat IgG (ref no. PK-6104; Vector Laboratories) in a total volume of 10 mL PBS/5% BSA. After washing the slides for 5 min with PBS/Triton (1.5 mL Triton X-100 per liter of 0.05 mol/L PBS), the slides were incubated with $\geq$200 µL of Pf39 rabbit antibody (1:200 dilution, diluted in blocking buffer) (*3*) at room temperature overnight. The antibody was produced in a rabbit by immunization with a peptide containing amino acids 34–

330 of the *P. falciparum* protein Pf39 (*4*,*5*) and was a kind gift from the laboratory of Prof. Gabriele Pradel (RWTH Aachen University, Germany). The next day, the slides were washed with PBS/Triton for 5 min and incubated with secondary antibody solution (Alexa Fluor 488 goat anti-rabbit, diluted 1:2000 in blocking buffer) for 1 h at room temperature. As a control, we performed the same labeling procedure for tissues by using only the secondary antibody. Then, the slides were washed with PBS/Triton 2× for 5 min each and, subsequently, 2× for 2 min each in PBS; they were placed in distilled water for 2 min and air dried. EUKITT mounting medium was used to fix the coverslips onto the glass slides containing our sections. The slides were imaged with a Leica DMI8 microscope (×100 oil objective, numerical aperture of 1.25). Slides were imaged by using the following exposure times: 500 ms (488 nm) and 9.8 ms (bright field) (both with gain of 5.9). Images were captured by using a Leica MC 120 HD camera. The images were examined and prepared for presentation by using a Fiji image processing package (https://fiji.sc) and Leica LAS X Office software.

Glycan Analysis

Glycan analysis for the Medici-clan mummy tissue was conducted at Asia-Pacific Glycomics Reference Site in Daejeon, Republic of Korea. The Medici-clan mummy tissue (≈10 mg) was cut into small pieces by using a razor blade and rehydrated for 72 h by using rehydration solution (100 mg of Na_2CO_3 in 10 mL of 30% ethanol). The rehydrated sample was centrifuged, and the pellet was separated from the supernatant and pulverized in 70% ethanol by using a tissue grinder. High molecular weight materials including glycoproteins were enriched from ground samples by using a mini dialysis unit (10-kDa molecular weight cutoff) and transferred to a microcentrifuge tube. Glycoproteins in the sample were denatured in a solution of NH_4HCO_3 and dithiothreitol at 95°C for 2 min. N-glycans were enzymatically released from denatured glycoproteins by using the glycosidase PNGase F at 37°C overnight and extracted from deglycosylated proteins by ethanol precipitation. N-glycans were further purified and enriched by using solid-phase extraction with a porous carbon cartridge. The eluate was dried by using vacuum centrifugation.

N-glycans released from Medici-clan mummy tissue were characterized by porous graphitized carbon-nano liquid chromatography chip/quadrupole time-of-flight mass spectrometry (MS) and MS/MS (Agilent 6530, Agilent Technologies) analysis with the

following parameters: positive ionization mode; VCap voltage, 1830 V; mass range (*m/z*), 300–2500 for MS and *m/z* of 100–3200 for MS/MS; acquisition time, 2 s/spectrum for MS and 0.63 s/spectrum for MS/MS; and collision-induced dissociation for MS/MS fragmentation. Raw MS data were computerized and processed by using the Molecular Feature Extractor algorithm included in the MassHunter Qualitative Analysis software (Agilent Technologies). Glycan searches used a list of glycan candidates comprised of monosaccharide combinations of hexose (Hex), N-acetylhexosamine (HexNAc), fucose (Fuc), N-acetyl-neuraminic acid (sialic acid), and xylose. To determine relative abundances of identified glycans, the chromatographic peak area for each glycan was integrated by using the ion intensity corresponding to the individual peak on the MS spectrum, and then relative abundance was calculated by using the equation: chromatogram peak area of individual glycan ÷ chromatogram peak area of all glycans × 100. Glycan isomers derived from porous graphitized carbon–liquid chromatography analysis were combined in a single glycan composition.

A total of 23 N-glycan compositions were identified by glycomic analysis of the Medici-clan mummy tissue (Appendix Table). Most N-glycans were pauci-mannose (n = 8, $Hex_{0–4}HexNAc_2Fuc_{0–1}$), fucosylated (n = 8, $Hex_{3–5}HexNAc_{3–6}Fuc_1$), high mannose (n = 4, $Hex_{5–9}HexNAc_2$), and truncated (n = 3, $Hex_{3–5}HexNAc_{3–4}$) types. N-glycans containing sialic acid and xylose were not detected in the mummy tissue sample. Pauci-mannose type N-glycans were the most abundant (47%) of total N-glycans. The relative abundances of N-glycans were 31% for fucosylated, 13% for high mannose, and 9% for truncated types (Appendix Figure 2, panel A). In a previous study, we found that N-glycans were preserved in mummy tissues over thousands of years through glycomic profiling of body tissue samples (*6*). Sialylated glycans were not observed in all mummies, whereas pauci-mannose, fucosylated, and truncated glycans were predominant in all mummy samples. Glycan profiling of Medici-clan mummy tissue was very similar to that in the previous study, which suggests that N-glycans found in Medici-clan mummy tissue might also have been degraded during mummification by exposure to the external environment and microbial activation, such as bacterial and fungal activation.

Our study focused on N-glycan analysis in the mummy tissue sample, but we also identified the presence of relatively abundant HexNAc polymers (n = 6, $HexNAc_{3–8}$) (Appendix Figure 2, panel B; Table). The relative proportion of HexNAc polymers (78%) was higher than

the overall relative abundance of N-glycan compounds (22%) in the Medici-clan mummy tissue (Appendix Figure 2, panel C). HexNAc polymers are not yet detectable in the human N-glycome, whereas those polymers are a primary type of cell envelope glycan found in a wide variety of fungi. The fungal cell wall is composed of highly cross-linked glycan polymers, such as chitins, mannoproteins, and glucans (glucose-derived polysaccharides) (*7*). Chitin is a repeating homopolymer of β1–4-linked N-acetylglucosamine that forms microfibrils by self-association; the chains are deposited on the cell wall of fungi (*8*).

Parasitic protozoa also express N-acetylglucosamine chitin polymers on their cell membrane. *Trichomonas vaginalis* and *Tritrichomonas fetus* parasites have surface-exposed chitin that might be part of a receptor for recognizing and binding to host cell lectins (*9*). The origin of HexNAc polymers found in the Medici-clan mummy tissue cannot be determined by only performing human N-glycan analysis. Therefore, we speculate that fungal microorganisms or parasitic protozoa could be the original source of HexNAc polymers (as chitin).

A unique glycan that might indicate B blood group antigen was found in the Medici-clan mummy tissue. Tandem MS spectrum of this unique glycan composition, $Hex_2HexNAc_2Fuc_1$ (*m/z* 895.3477, z = 1), was interpretated (Appendix Figure 2, panel D**)**. The fragment peaks observed at *m/z* 674.24 indicated reducing end HexNAc fragmentation from the precursor ion and assigned with Hex_2–nonreducing $HexNAc_1Fuc_1$ that is characteristic of a B antigen-carrying structure. Additional investigation of glycans including blood group epitopes is needed to determine the blood type of the Medici-clan mummy. However, the unique glycan identified in our study indicates the possibility of blood type identification through glycan analysis of mummy tissue.

DNA-Based Analysis

Molecular analysis of the Medici tissue sample was performed at the ancient DNA laboratory at Eurac Research, Institute for Mummy Studies in Bolzano, Italy. Sample documentation, preparation, and DNA extraction were performed in a dedicated pre-PCR area that requires strict procedures for studies of ancient DNA: use of protective clothing, UV-light exposure of the equipment and bleach sterilization of surfaces, use of PCR workstations, and filtered pipette tips. DNA was extracted from 200 mg soft tissue by using an organic solvent-based extraction method, including a DNA precipitation step with linear polyacrylamide (*10*). A

DNA extraction blank was included in the extraction process. Both the extracted DNA and extraction blank were first subjected to a highly sensitive *P. falciparum*–specific PCR assay that targets the *stevor* gene family (*11*) and has been previously used for ancient human remains (*12*). We were unable to amplify the 188-bp PCR fragment, most likely because of the high degree of DNA fragmentation observed in our sample. In parallel to the PCR assay, we transformed the DNA extract into a double-indexed library for subsequent Illumina multiplex sequencing (*13*,*14*). A library preparation control (PCR-grade water) was included. The libraries of the extraction blank and library preparation control were negative. The library of the tissue sample was subjected to a paired-end shotgun sequencing approach on an Illumina HiSeq2500 platform (Illumina Corp., https://www.illumina.com) by using 2 × 101 cycles and HiSeq v3 chemistry following the manufacturer's protocol for multiplex sequencing. Sequence data are available from the European Nucleotide Archive (https://www.ebi.ac.uk/ena) under project accession no. PRJEB61275.

The metagenomic shotgun dataset was subjected to a bioinformatic pipeline to identify traces of endogenous human and parasite DNA. Illumina reads were first quality-checked by using FastQC (http://www.bioinformatics.babraham.ac.uk/projects/fastqc) and then subjected to adaptor removal and read merging by using the SeqPrep tool version 1.2 (https://github.com/jstjohn/SeqPrep). Pre-processed shotgun reads were first aligned to the full human genome (build Hg19, default mapping parameters) (*15*) by using Bowtie 2 version 1.2.1.1 and the end-to-end parameter (*16*). Deduplication of the mapped reads was performed by using the DeDup tool version 0.12.8 (https://github.com/apeltzer/DeDup). Both minimum mapping and base quality were set to 30. The binary alignment/map file was checked for characteristic ancient DNA nucleotide misincorporation frequency patterns by using mapDamage2 version 2.0.9 (*17*). The genetic sex was assigned by using a maximum-likelihood method on the basis of the karyotype frequency of the mapped human X and Y chromosome reads (*18*). Human DNA analysis revealed that only 0.06% of all reads aligned to the human genome. The mapped reads were highly fragmented and displayed DNA damage. The sex of the Medici family member who supplied the tissue was assigned with low confidence as male. The low genome coverage did not allow any further authentication, such as a contamination check using Schmutzi (*19*) or comparative analysis of mitochondrial haplogroup assignment using HaploGrep2 (*20*).

Shotgun reads were aligned against the full genome assemblies of *P. falciparum* (GenBank assembly no. GCA_000002765) by using Burrows-Wheeler Aligner software (*21*) and previously described parameters (*22*). The mapped data was subjected to the same deduplication and quality filtering as described for human DNA analysis. Subsequently, a sequence similarity search of all mapped reads against the *P. falciparum* genome by using blastn (*23*) and the complete NCBI nucleotide database (*24*) was performed. Blast results were taxonomically assigned by using MEGAN6 and the LCA (Lowest Common Ancestor) algorithm (*25*). Thereby, only 2 of 36,406 mapped reads were unambiguously assigned to *P. falciparum* (when this assignment appeared as a top blast hit). Finally, we assessed a general taxonomic profile of the sequencing reads by using DIAMOND blastx search (*26*) against the RefSeq non-redundant protein database (http://www.ncbi.nlm.nih.gov/RefSeq). The DIAMOND tables were converted to rma6 (blast2rma tool) format (–minPercentIdentity 97), imported into MEGAN6 software (*25*), and subsequently visualized by using the Krona tool (*27*). Most reads in the samples were assigned to bacteria; <6% reads were eukaryotic, and ≤98% of those were fungi (Appendix Figure 2, panel E).

References

1. Mekota AM, Vermehren M. Determination of optimal rehydration, fixation and staining methods for histological and immunohistochemical analysis of mummified soft tissues. Biotech Histochem. 2005;80:7–13. PubMed https://doi.org/10.1080/10520290500051146

2. Romeis-Mikroskopische Techniken. Mulisch M, Welsch U, editors. 18th ed. Berlin: Springer Spektrum; 2010.

3. Bennink S, von Bohl A, Ngwa CJ, Henschel L, Kuehn A, Pilch N, et al. A seven-helix protein constitutes stress granules crucial for regulating translation during human-to-mosquito transmission of *Plasmodium falciparum*. PLoS Pathog. 2018;14:e1007249. PubMed https://doi.org/10.1371/journal.ppat.1007249

4. Hanhsen B, Farrukh A, Pradel G, Ngwa CJ. The *Plasmodium falciparum* CCCH zinc finger protein ZNF4 plays an important role in gametocyte exflagellation through the regulation of male enriched transcripts. Cells. 2022;11:1666. PubMed https://doi.org/10.3390/cells11101666

5. Scholz SM, Simon N, Lavazec C, Dude M-A, Templeton TJ, Pradel G. PfCCp proteins of *Plasmodium falciparum*: gametocyte-specific expression and role in complement-mediated inhibition of exflagellation. Int J Parasitol. 2008;38:327–40. PubMed https://doi.org/10.1016/j.ijpara.2007.08.009

6. Ozcan S, Kim BJ, Ro G, Kim J-H, Bereuter TL, Reiter C, et al. Glycosylated proteins preserved over millennia: N-glycan analysis of Tyrolean Iceman, Scythian Princess and Warrior. Sci Rep. 2014;4:4963. PubMed https://doi.org/10.1038/srep04963

7. Masuoka J. Surface glycans of *Candida albicans* and other pathogenic fungi: physiological roles, clinical uses, and experimental challenges. Clin Microbiol Rev. 2004;17:281–310. PubMed https://doi.org/10.1128/CMR.17.2.281-310.2004

8. Colley KJ, Varki A, Kinoshita T. Cellular organization of glycosylation. In: Varki A, Cummings RD, Esko JD, Stanley P, Hart GW, Aebi M, et al., editors. Essentials of glycobiology. New York: Cold Spring Harbor Laboratory Press; 2015. p. 41–9.

9. Kneipp LF, Andrade AF, de Souza W, Angluster J, Alviano CS, Travassos LR. Trichomonas vaginalis and Tritrichomonas foetus: expression of chitin at the cell surface. Exp. Parasitol. 1998;89:195–204. PubMed https://doi.org/10.1006/expr.1998.4290

10. Maixner F, Mitterer C, Jäger HY, Sarhan MS, Valverde G, Lücker S, et al. Linear polyacrylamide is highly efficient in precipitating and purifying environmental and ancient DNA. Methods Ecol Evol. 2022;13:653–67. https://doi.org/10.1111/2041-210X.13772

11. Cheng Q, Lawrence G, Reed C, Stowers A, Ranford-Cartwright L, Creasey A, et al. Measurement of Plasmodium falciparum growth rates in vivo: a test of malaria vaccines. Am J Trop Med Hyg. 1997;57:495–500. PubMed https://doi.org/10.4269/ajtmh.1997.57.495

12. Hawass Z, Gad YZ, Ismail S, Khairat R, Fathalla D, Hasan N, et al. Ancestry and pathology in King Tutankhamun's family. JAMA. 2010;303:638–47. PubMed https://doi.org/10.1001/jama.2010.121

13. Kircher M, Sawyer S, Meyer M. Double indexing overcomes inaccuracies in multiplex sequencing on the Illumina platform. Nucleic Acids Res. 2012;40:e3. PubMed https://doi.org/10.1093/nar/gkr771

14. Meyer M, Kircher M. Illumina sequencing library preparation for highly multiplexed target capture and sequencing. Cold Spring Harb Protoc. 2010;2010:pdb.prot5448. PubMed https://doi.org/10.1101/pdb.prot5448

15. Rosenbloom KR, Armstrong J, Barber GP, Casper J, Clawson H, Diekhans M, et al. The UCSC genome browser database: 2015 update. Nucleic Acids Res. 2015;43:D670–81. PubMed https://doi.org/10.1093/nar/gku1177

16. Langmead B, Salzberg SL. Fast gapped-read alignment with Bowtie 2. Nat Methods. 2012;9:357–9. PubMed https://doi.org/10.1038/nmeth.1923

17. Jónsson H, Ginolhac A, Schubert M, Johnson PLF, Orlando L. mapDamage2.0: fast approximate Bayesian estimates of ancient DNA damage parameters. Bioinformatics. 2013;29:1682–4. PubMed https://doi.org/10.1093/bioinformatics/btt193

18. Skoglund P, Storå J, Götherström A, Jakobsson M. Accurate sex identification of ancient human remains using DNA shotgun sequencing. J Archaeol Sci. 2013;40:4477–82. https://doi.org/10.1016/j.jas.2013.07.004

19. Renaud G, Slon V, Duggan AT, Kelso J. Schmutzi: estimation of contamination and endogenous mitochondrial consensus calling for ancient DNA. Genome Biol. 2015;16:224. PubMed https://doi.org/10.1186/s13059-015-0776-0

20. Weissensteiner H, Pacher D, Kloss-Brandstätter A, Forer L, Specht G, Bandelt HJ, et al. HaploGrep 2: mitochondrial haplogroup classification in the era of high-throughput sequencing. Nucleic Acids Res. 2016;44:W58-63. PubMed https://doi.org/10.1093/nar/gkw233

21. Li H, Durbin R. Fast and accurate long-read alignment with Burrows-Wheeler transform. Bioinformatics. 2010;26:589–95. PubMed https://doi.org/10.1093/bioinformatics/btp698

22. Marciniak S, Prowse TL, Herring DA, Klunk J, Kuch M, Duggan AT, et al. *Plasmodium falciparum* malaria in 1st–2nd century CE southern Italy. Curr Biol. 2016;26:R1220–2. PubMed https://doi.org/10.1016/j.cub.2016.10.016

23. Altschul SF, Gish W, Miller W, Myers EW, Lipman DJ. Basic local alignment search tool. J Mol Biol. 1990;215:403–10. PubMed https://doi.org/10.1016/S0022-2836(05)80360-2

24. NCBI Resource Coordinators. Database resources of the National Center for Biotechnology Information. Nucleic Acids Res. 2018;46:D8–13. PubMed https://doi.org/10.1093/nar/gkx1095

25. Huson DH, Beier S, Flade I, Górska A, El-Hadidi M, Mitra S, et al. MEGAN community edition—interactive exploration and analysis of large-scale microbiome sequencing data. PLoS Comput Biol. 2016;12:e1004957. PubMed https://doi.org/10.1371/journal.pcbi.1004957

26. Buchfink B, Xie C, Huson DH. Fast and sensitive protein alignment using DIAMOND. Nat Methods. 2015;12:59–60. PubMed https://doi.org/10.1038/nmeth.3176

27. Ondov BD, Bergman NH, Phillippy AM. Interactive metagenomic visualization in a web browser. BMC Bioinformatics. 2011;12:385. PubMed https://doi.org/10.1186/1471-2105-12-385

Appendix Table. A list of glycans identified from the Medici-clan mummy tissue*

Sample no.	Mass†	Composition				Type	Relative abundance (%)
		Hex	HexNAc	Fuc	NeuAc		
N-glycans							
1	424.169	0	2	0	0	Pauci-mannose	2.87
2	586.222	1	2	0	0	Pauci-mannose	8.96
3	732.280	1	2	1	0	Pauci-mannose	4.66
4	748.275	2	2	0	0	Pauci-mannose	7.88
5	894.333	2	2	1	0	Pauci-mannose	2.15
6	910.328	3	2	0	0	Pauci-mannose	10.79
7	1056.386	3	2	1	0	Pauci-mannose	3.81
8	1072.381	4	2	0	0	Pauci-mannose	6.14
9	1259.465	3	3	1	0	Fucosylated	9.54
10	1462.544	3	4	1	0	Fucosylated	8.55
11	1665.624	3	5	1	0	Fucosylated	2.64
12	1868.703	3	6	1	0	Fucosylated	3.18
13	1624.597	4	4	1	0	Fucosylated	1.61
14	1827.677	4	5	1	0	Fucosylated	2.37
15	1786.650	5	4	1	0	Fucosylated	2.06
16	1989.729	5	5	1	0	Fucosylated	0.85
17	1234.433	5	2	0	0	High mannose	5.87
18	1396.486	6	2	0	0	High mannose	1.21
19	1558.539	7	2	0	0	High mannose	2.69
20	1720.592	8	2	0	0	High mannose	3.40
21	1113.407	3	3	0	0	Truncated	1.34
22	1275.460	4	3	0	0	Truncated	3.27
23	1640.592	5	4	0	0	Truncated	4.16
HexNAc polymers							
1	627.249	0	3	0	0	HexNAc polymer	23.40
2	830.328	0	4	0	0	HexNAc polymer	17.38
3	1033.408	0	5	0	0	HexNAc polymer	21.46
4	1236.487	0	6	0	0	HexNAc polymer	23.43
5	1439.566	0	7	0	0	HexNAc polymer	9.29
6	1642.646	0	8	0	0	HexNAc polymer	5.03

*Fuc, fucose; Hex, hexose; HexNAc, N-acetylhexosamine; NeuAc, N-acetyl-neuraminic acid.
†Mass units are m/z; ratio of ion mass (m) to ion charge (z).

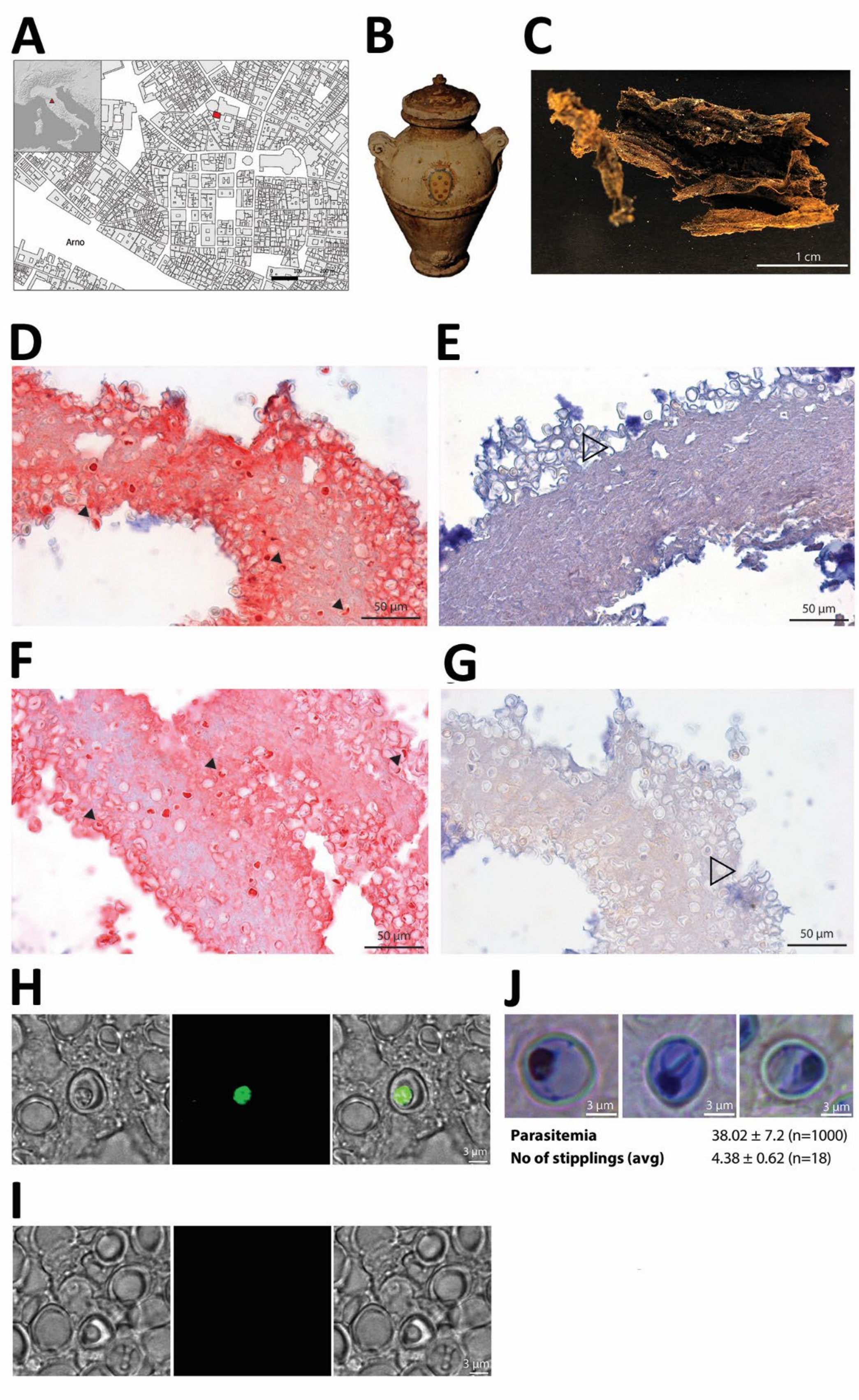
A
B
C
Arno
1 cm
D
E
50 µm
F
G
50 µm
H
3 µm
I
3 µm
J
3 µm
Parasitemia 38.02 ± 7.2 (n=1000)
No of stipplings (avg) 4.38 ± 0.62 (n=18)

Appendix Figure 1. Microscopic investigation of visceral tissue from the Medici family. A) Location of the San Lorenzo Basilica in the center of Florence, Italy. Geographic location of Florence in Italy (red triangle, upper left map). Location of San Lorenzo Basilica (red box) in the Florence city center. B) Terracotta jar where internal organs of a Medici family member were placed. The Medici coat of arms is visible on the jar. C) Medici tissue (ID 1297) from 1 jar that was subjected to microscopic and molecular analysis. D) Immunohistochemistry of a paraffin section of tissue labeled with an antibody against *Plasmodium* spp.–specific aldolase protein. Arrows show positive staining of parasites in erythrocytes. E) Isotype control for the aldolase-specific antibody. No staining was observed in erythrocytes (arrow). F) Immunohistochemistry of paraffin section of tissue labeled with an antibody against *Plasmodium falciparum*–specific histidine-rich protein. Arrows show positive staining of erythrocytes. G) Isotype control for histidine-rich protein-specific antibody. No staining was observed in erythrocytes (arrow). H) Immunofluorescence analysis of paraffin section of tissue labeled with antibody against Pf39, an endoplasmic reticulum resident protein of *P. falciparum*. I) Secondary antibody control conducted during immunolabelling with antibody against Pf39. No staining was observed in erythrocytes. J) Staining of sectioned paraffin-embedded tissue with buffered Giemsa (pH 6.8) revealed stippled structures that resemble Maurer's clefts. These unique structures can be observed in the cytoplasm of malaria-parasitized erythrocytes where they play a role in protein trafficking. However, additional putative functions might be conducted by these organelles as well.

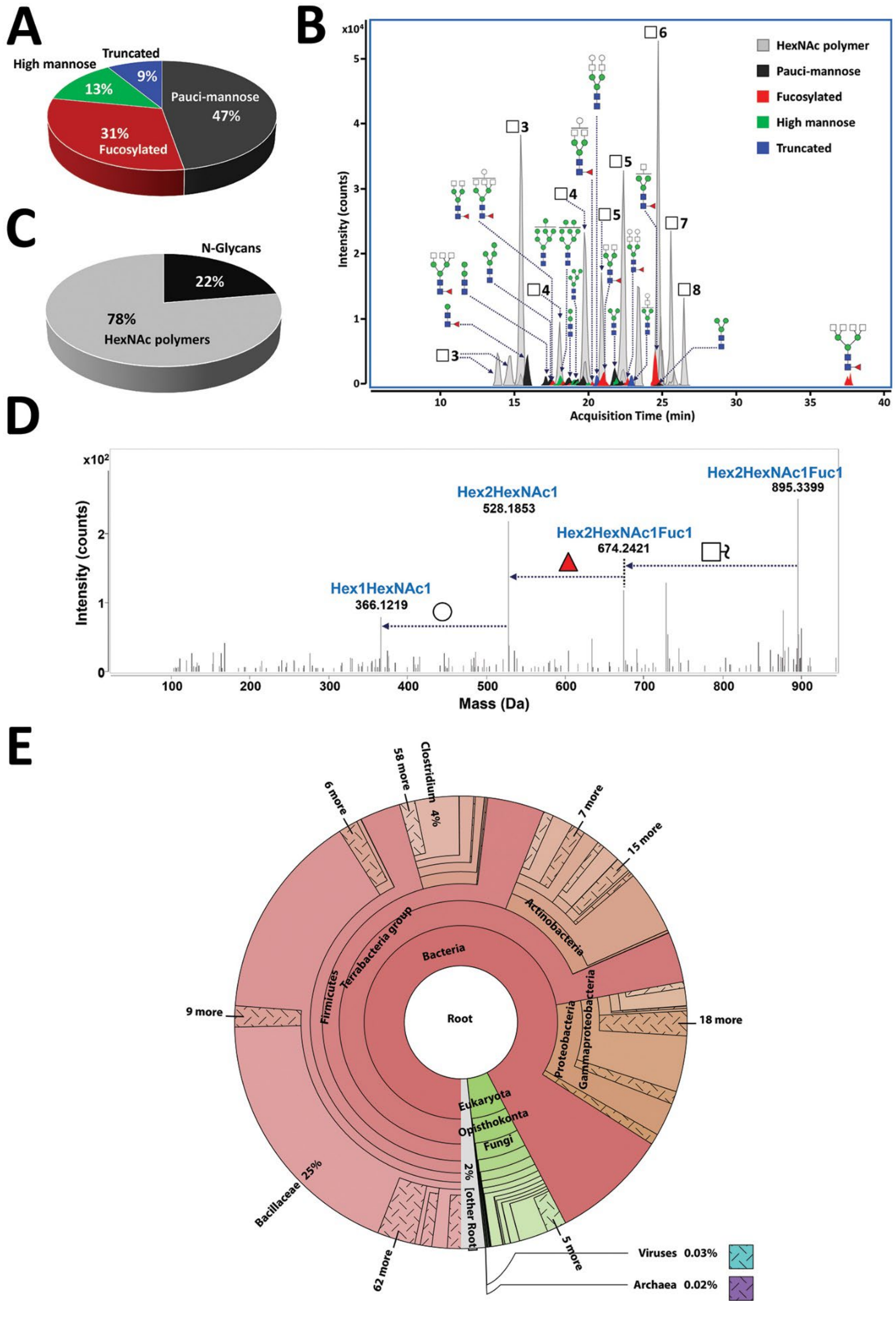

A
Truncated
High mannose
9%
13%
Pauci-mannose
47%
31%
Fucosylated
B
x10⁴
HexNAc polymer
Pauci-mannose
Fucosylated
High mannose
Truncated
Intensity (counts)
Acquisition Time (min)
C
N-Glycans
22%
78%
HexNAc polymers
D
x10²
Hex2HexNAc1
528.1853
Hex2HexNAc1Fuc1
895.3399
Hex2HexNAc1Fuc1
674.2421
Hex1HexNAc1
366.1219
Intensity (counts)
Mass (Da)
E
58 more
Clostridium 4%
6 more
7 more
15 more
Actinobacteria
Terrabacteria group
Bacteria
Firmicutes
9 more
Root
Proteobacteria
Gammaproteobacteria
18 more
Eukaryota
Opisthokonta
Fungi
2% [other Root]
Bacillaceae 25%
62 more
5 more
Viruses 0.03%
Archaea 0.02%

Appendix Figure 2. Glycan and metagenomics analyses of the Medici tissue. (A) Pie chart indicating relative abundances of Medici-clan mummy N-glycans grouped according to biosynthetic classes: pauci-mannose (dark grey), fucosylated (red), high mannose (green), and truncated (blue). (B) Extracted compound chromatograms of N-glycans and N-acetylhexosamine (HexNAc) polymers identified from the Medici-clan mummy tissue by porous graphitized carbon-nano liquid chromatography chip/quadrupole time-of-flight mass spectrometry acquisition. Putative structures of abundant major peaks have been labeled. Glycan symbols are: white circle, hexose; green circle, mannose; white square, N-acetylhexosamine; blue square, N-acetylglucosamine; and red triangle, fucose. C) Pie chart for relative abundances of N-glycans and HexNAc polymers in the Medici-clan mummy tissue. D) Tandem mass spectrometry spectrum of Hex2HexNAc2Fuc1, m/z 895.3477 (z = 1), [M+H]+ identified from the Medici-clan mummy tissue. Glycan symbols: white circle, hexose; white square, N-acetylhexosamine; and red triangle, fucose. (E) Taxonomic overview of the DNA sequence reads in the shotgun datasets of the Medici sample 1297. The metagenomic reads were taxonomically assigned by using the Diamond tool (*26*) against the NCBI RefSeq non-redundant protein database (http://www.ncbi.nlm.nih.gov/RefSeq).
